# Portable Lab for Shipping (POLS): A Biosensor-Based System for Rapid Onboard Detection of *Escherichia coli* and *Enterococcus* spp. in Ballast Water

**DOI:** 10.3390/microorganisms13122878

**Published:** 2025-12-18

**Authors:** Stephanie Agioti, Emmanouil Loulakis, Lazaros Konstantinou, Eleni Varda, Antonios Inglezakis, Konstantinos Loizou, Theofylaktos Apostolou, Agni Hadjilouka

**Affiliations:** 1 Department of Life Sciences, School of Sciences, European University Cyprus, 2404 Nicosia, Cyprus; stephanie.agioti@gmail.com (S.A.); manoskriti2000@gmail.com (E.L.); 2 EMBIO Diagnostics Ltd., Athalassas, 2018 Nicosia, Cyprus; lazaros.konstantinou@embiodiagnostics.eu (L.K.); e.varda@embiodiagnostics.eu (E.V.); a.inglezakis@embiodiagnostics.eu (A.I.); k.loizou@embiodiagnostics.eu (K.L.); theo.apo@embiodiagnostics.eu (T.A.)

**Keywords:** ballast water, microbiological water quality, *Escherichia coli*, *Enterococcus* spp., International Maritime Organization, potentiometry

## Abstract

Ballast water (BW) is a major pathway for the spread of invasive microorganisms and pathogens, posing significant ecological and public health risks. The International Maritime Organization (IMO) has established strict discharge standards, yet routine monitoring remains limited, and no reliable onboard test is currently available to assist crews in verifying BW quality before discharge. This study presents the development of a rapid, portable method for onboard microbiological assessment of BW, based on potentiometric detection and biosensors engineered with the Bioelectric Recognition Assay (BERA). Two complementary approaches were evaluated: (i) direct potentiometric measurements of contaminated and non-contaminated samples, which confirmed the feasibility of detecting microbial presence but were restricted by high detection limits, and (ii) development of biosensors specifically engineered for *Escherichia coli* and *Enterococcus* spp. to improve specificity and lower the limit of detection (LOD). Results demonstrated successful detection of both microorganisms, with performance characteristics of 83.3% sensitivity and 81.9% accuracy for *Enterococcus* spp. (LOD: 10^2^ CFU 100 mL^−1^), and 89.8% sensitivity and 85.1% accuracy for *Escherichia coli* (LOD: 250 CFU 100 mL^−1^). These findings underscore the potential of biosensor-based systems as practical, crew-operated tools for early warning and real-time monitoring of ballast water quality, supporting compliance with IMO standards and contributing to safer, more sustainable maritime operations.

## 1. Introduction

Maritime trade remains the dominant mode of transporting goods globally. Over 80% of international trade by volume occurs via oceans, with ships transporting an estimated 11.6–12.3 billion tons of cargo annually as of 2023 [1]. While ballast water (BW) is essential for vessel safety, it also poses serious ecological, economic, and public health risks due to the unintentional transport of marine organisms. BW refers to water taken from coastal areas at ports and stored in designated tanks aboard ships. It is crucial for ensuring a vessel’s stability and maintaining proper displacement, especially when the ship is lightly loaded or empty. Additionally, BW can be used to increase the ship’s draft, allowing it to submerge further when needed, such as when navigating under bridges [2]. As a ship’s weight changes frequently during loading and unloading at various ports, BW must be pumped in or discharged to maintain safe stability conditions [3].

The uptake and discharge of BW can introduce non-native organisms into marine ecosystems, a process identified by the International Maritime Organization (IMO) as one of the greatest global threats to marine biodiversity. BW may carry thousands of aquatic microbes, plants, and animals, which can establish themselves in new environments and cause widespread ecological damage. Although most microorganisms in BW are harmless, studies have identified dozens of potentially pathogenic species, including *Vibrio*, *Pseudomonas*, *Acinetobacter*, *Escherichia coli* (*E. coli*), and *Staphylococcus aureus*, in samples worldwide [4,5].

To mitigate these risks, the IMO adopted the BW Management Convention in 2004, which remains in force [6]. Under this agreement, all ships on international voyages must treat their BW before discharge in accordance with the approved standards. Ships are also required to maintain a BW Record Book and carry an International Ballast Water Management (IBWM) Certificate. Discharging untreated BW that does not meet IMO standards [7] can result in substantial financial penalties [8].

Despite widespread adoption of BW treatment systems, port inspections frequently report non-compliance. Currently, testing is performed exclusively by Port State Control (PSC) authorities, as ships lack onboard analytical capability. Implementing such onboard testing would allow crews to verify treatment efficiency in real time and take corrective action when necessary [9]. However, full compliance testing generally requires complex sampling procedures and the assistance of trained specialists to support PSC officers during inspections.

The IMO has set strict microbiological discharge limits for three key indicator organisms: *E. coli*, intestinal *Enterococci*, and *Vibrio cholerae* (*V. cholerae*). According to the D-2 standard, *V. cholerae* must be absent from 100 mL, *E. coli* must be less than 250 CFU 100 mL^−1^, and intestinal *Enterococci* must remain below 100 CFU 100 mL^−1^ [10].

In addition to serving as regulatory indicators, these microorganisms also pose direct health risks. *E. coli*, while a normal inhabitant of the human gut, includes pathogenic strains capable of causing gastrointestinal illness, pneumonia, and hemolytic–uremic syndrome, and is widely used as a marker of fecal contamination [11,12]. Intestinal *Enterococci*, primarily *Enterococcus faecalis* and *Enterococcus faecium*, are recognized as important indicators of fecal contamination, exhibiting notable antibiotic resistance, which complicates the treatment of enterococcal infections [13]. *V. cholerae*, particularly the O1 and O139 serogroups, can cause cholera, a severe diarrheal disease responsible for thousands of deaths annually [14,15].

To comply with regulations, reliable monitoring methods are critical. Traditional culture-based techniques remain the gold standard, offering both qualitative and quantitative results. However, these methods require incubation periods of several days before definitive results are available [16,17]. Flow cytometry offers faster analysis but requires expensive laboratory equipment and highly trained personnel to perform the tests [18,19]. In addition, molecular techniques like Polymerase Chain Reaction (PCR) and Nucleic Acid-Based Sequence Amplification (NASBA) have revolutionized BW pathogen detection. They enable rapid, culture-free identification of organisms such as *E. coli* and *V. cholerae* with high sensitivity [20]. Despite these advantages, these methods require specialized equipment, trained personnel, and controlled laboratory conditions, which limit their feasibility for routine onboard use. Similarly, while advanced technologies such as photonic immunosensors, nanostructure enhancement, and impedance-based microchips achieve low detection limits, they lack the ruggedness, simplicity, and rapid deployment necessary for maritime environments [21,22].

This underscores the urgent need for simple, field-deployable methods capable of providing real-time onboard detection. To address these limitations, portable indicative testing kits have been developed for use directly on ships. Examples include marine potable water kits for coliform and *E. coli* detection, commercially available from Parker Kittiwake and DTK Water. These rely on dipslides that exhibit visible color changes under fluorescence or UV illumination after an incubation period of 24 h, indicating bacterial presence [23,24]. Similarly, the Vermicon’s Scan VIT^®^ *E. coli*/*Enterococci* test kit, which applies a FISH-based approach, yields results within 12 h [25]. Another portable option, the Luminultra B-QUA system, utilizes ATP-based detection to estimate cellular ATP (cATP) activity, offering semi-quantitative indications such as most likely compliant, close to limit, or most likely non-compliant within approximately 1 h [26]. Collectively, these existing tools provide only indicative results and require either lengthy incubation or specialized handling, highlighting the persistent gap between laboratory accuracy and operational feasibility. Therefore, the development of a new, rapid, and user-friendly method capable of delivering real-time microbial detection directly onboard is both necessary and timely.

**Figure 1:**
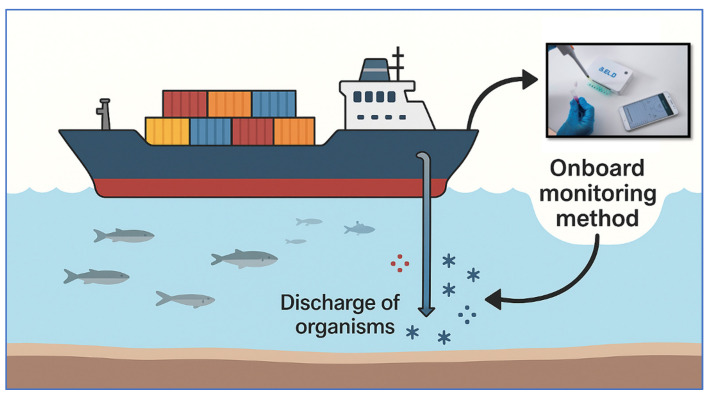
Real-Time Onboard Monitoring of Ballast Water Discharges in Global Shipping: IMO D-2 Compliance and the Detection of *E. coli*, intestinal *Enterococci* and *V. cholerae* (AI-generated illustration). The (*) symbol and the red/blue colored dots represent microorganisms discharged into the sea and detected by the new method.

To address this gap, this study developed a real-time, portable method for evaluating the microbiological quality of BW before uptake. The goal was to provide crews with an early-warning system capable of identifying microbial concentrations exceeding regulatory thresholds, thus prompting timely treatment. A portable potentiometric device (EMBIO Diagnostics Ltd., Nicosia, Cyprus) was used to monitor changes in electric potential, offering intuitive, real-time readings aligned with IMO standards (Figure 1).

## 2. Materials and Methods

### 2.1. Materials and Reagents

Monkey African green kidney (Vero) cell cultures were provided from LGC Promochem (Teddington, UK). Fetal bovine serum (FBS), antibiotics (streptomycin–penicillin), L-glutamine and L-alanine, and trypsin/EDTA (ethylenediaminetetraacetic acid) were purchased from Sigma-Aldrich (Taufkirchen, Germany). Monoclonal antibodies against *E. coli* [anti-*E.coli* antibody, ABIN3027591], *Enterococcus* spp. [anti-*Enterococcus* antibody, ABIN3027582] and *V. cholerae* [anti-*V. cholerae* ABIN344551] were purchased from Antibodies online (Limerick, PA, USA). Sodium chloride was purchased from Merck (Darmstadt, Germany), Brain Heart Infusion (BHI) from Biolife (Milan, Italy), Harlequin UTI Chromogenic Agar from Neogen (Lansing, MI, USA) and Thiosulfate Citrate Bile Salts sucrose (TCBS) from Neogen (Lansing, MI, USA).

### 2.2. Study Design

The study was structured and conducted in two phases. In the first phase, direct potentiometric measurements were performed on contaminated and non-contaminated samples. Specifically, *E. coli* and *Enterococcus* spp. (i.e., *E. faecium* and *E. faecalis*) were tested at four different concentrations (10^2^, 10^3^, 10^5^, and 10^7^ CFU 100 mL^−1^), along with control samples of ballast water. Each sample first underwent a pre-filtration step using a 10 μm pore size filter (Macherey-Nagel, Düren, German) to remove larger eukaryotic microorganisms, such as protozoa. The pre-filtered water was collected in sterile Duran glass containers. Each sample was then contaminated with different concentrations of the under-study bacteria (100 mL in total) and then passed through a sterile cellulose ester membrane filter (47 mm diameter, 0.45 μm pore size, Cytiva, Buckinghamshire, UK), which retained the bacterial cells. The filters were then transferred into 2 mL buffer solution to release microorganisms for B.EL.D^TM^ analysis. The same filtration steps were followed also for the non-contaminated samples (control samples).

**Figure 2:**
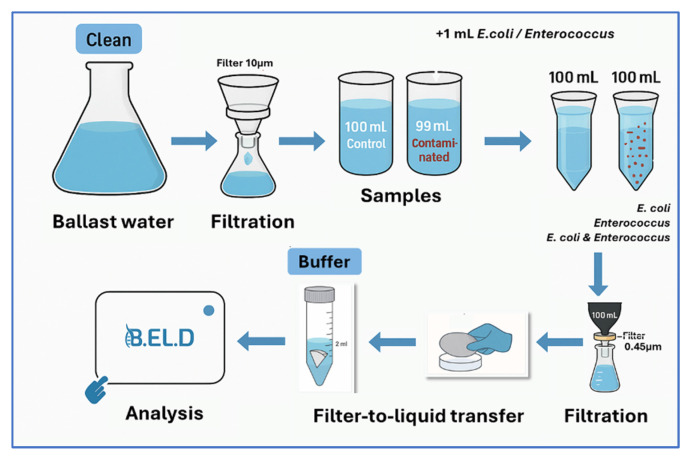
Testing procedure of the newly developed method (AI-generated illustration).

In the second phase, specific biosensors targeting *E. coli* and *Enterococcus* spp. were developed to eliminate the limitations of the first phase’s experiments. The filtration procedure followed the same protocol as in phase one; however, in the second phase, the concentrations of the microorganisms were adjusted at lower levels. The selected concentrations for each bacterium were determined according to their respective IMO threshold limits. The chosen concentrations were 250, 500, 10^3^, and 10^4^ CFU 100 mL^−1^ for *E. coli*, and 10^2^, 500, 10^3^, and 10^4^ CFU 100 mL^−1^ for *Enterococcus* spp. Figure 2 is a schematic representation of the filtration and testing procedure of the new method.

### 2.3. Cell Culture and Biosensor Development

African green monkey kidney (Vero) cells were used throughout the study, as they constitute the established cellular platform for BERA biosensors. Their stable membrane composition, ease of antibody electroinsertion, and ability to generate robust and reproducible electrical responses make them highly suitable for this application. The use of Vero cells in BERA-based pathogen and chemical detection has been extensively validated in previous studies [27,28,29]. In this work, the cells were maintained following the previously described procedure by Apostolou et al. [27]. Briefly, the cells were grown in Dulbecco’s Modified Eagle Medium (DMEM) supplemented with 10% fetal bovine serum (FBS), 10% streptomycin/penicillin, and 10% L-glutamine and L-alanine. Cultures were incubated at 37 °C in a humidified atmosphere containing 5% CO_2_. To detach the cells, trypsin/EDTA solution was added and incubated for 10 min at 37 °C. Membrane-engineered cells were then produced, using the BERA method, according to previously established protocols [28,29]. Specifically, the harvested cell pellet was resuspended in phosphate-buffered saline containing 5 μg mL^−1^ of either the anti-*E. coli*, or the anti-*Enterococcus* antibodies and incubated in ice for 20 min. The anti-*E. coli* antibody is a goat polyclonal antibody raised against multiple *E. coli* serotypes and is specific to a broad range of *E. coli* strains. The anti-*Enterococcus* antibody is a rabbit polyclonal antibody raised against whole *Enterococcus faecium* cells and reported as reactive with whole *Enterococcus* species, with possible limited cross-reactivity to related microbes due to its unabsorbed nature. Both antibodies were >95% purified and validated for microbial detection applications. The resulting cell–antibody suspension was placed in electroporation cuvettes, and electroinsertion was performed by applying two square-wave electric pulses at 1800 V/cm using an Eppendorf Eporator (Hamburg, Germany). The transfected cells were subsequently incubated in nutrient medium at 37 °C with 5% CO_2_ for 24 h. After incubation, the medium was discarded, and the Vero/anti-[*E. coli* or *Enterococcus*] cells were mechanically detached and collected in nutrient medium into Eppendorf tubes.

### 2.4. Bacteria Culturing and Sensor Fabrication from Vero Cells

The study utilized *Escherichia coli* ATCC 10536, *Enterococcus faecalis* ATCC 29212, and *Enterococcus faecium* ATCC 700221, and *Vibrio cholerae* ATCC 9459. Although the legislation refers to intestinal *enterococci* in general, *E. faecalis* and *E. faecium* were used throughout this study as representative species for practical and scientific reasons, as they are the most prevalent and well-characterized members of this group and are widely employed as model microorganisms.

The bacterial strains were stored at −20 °C in nutrient broth containing 50% glycerol. Before use, each strain was cultured twice in BHI broth at 37 °C for 24 h. Experimental assays were conducted using cultures of *E. coli*, *Enterococcus* spp. and *V. cholerae*, with concentrations of 8 log CFU mL^−1^ after 24 h incubation at 37 °C. Before each experiment, cultures were centrifuged for 15 min at 3500 rpm at 4 °C, washed twice with MRD diluent, and resuspended in the same diluent. Then successive dilutions were performed to achieve the expected concentrations.

During the first phase, samples were analyzed potentiometrically (without the use of biosensors) to establish baseline responses. Four concentrations of each bacterium were tested—10^2^, 10^3^, 10^5^, and 10^7^ CFU 100 mL^−1^—along with a control sample. In the second experimental phase, the focus shifted toward evaluating the IMO regulatory thresholds, which were set at 0 CFU 100 mL^−1^ for *V. cholerae*, 100 CFU 100 mL^−1^ for *Enterococcus* spp. and 250 CFU 100 mL^−1^ for *E. coli*. The upper permissible limits defined by the IMO for these microorganisms were included, since it was anticipated that the biosensor-based detection method could enable the reliable identification of considerably lower microbial concentrations. Specifically, for *Enterococcus* spp., samples were prepared at control levels and at 10^2^, 500, 10^3^, and 10^4^ CFU 100 mL^−1^, while for *E. coli*, samples included control and concentrations of 250, 500, 10^3^, and 10^4^ CFU 100 mL^−1^. Organism presence or absence was evaluated using UTI—a specific culture media in which *E. coli* is shown by red colonies and *Enterococcus* spp. by turquoise colonies.

Due to legislation mandating the absence of *V. cholerae* in 100 mL of BW, preliminary studies were conducted to investigate the feasibility of the new method to detect the pathogen at very low population levels (≈10 CFU mL^−1^). The results indicated that the detection of the microorganism was not feasible at such concentrations. Considering the importance of sample enrichment for the detection of microorganisms whose regulatory limits require their complete absence, no further investigation was pursued for this microorganism, as higher detection limits would have had no practical relevance under the current regulatory framework. Therefore, none of the results related to this bacterium are presented in this study.

### 2.5. Assay Procedure

All assays were performed using the B.EL.D™ device, a portable device developed by EMBIO Diagnostics Ltd. in Strovolos, Cyprus. This multichannel potentiometric instrument is equipped with high-precision analog-to-digital converters capable of detecting bioelectric signals from cellular biorecognition elements. These elements are deposited onto eight channel disposable screen-printed electrodes (SPEs) connected through a replaceable underside connector, enabling simultaneous multichannel analysis. When combined with BERA technology, the device enables accurate, rapid, and high throughput testing of samples. As Kintzios and collaborators previously described, the Bioelectric Recognition Assay (BERA) (ImmuSmol, Strasbourg, France) is a cell-based biosensing technique in which membrane-engineered, antibody-functionalized living cells generate quantifiable alterations in transmembrane potential upon selective analyte binding. These bioelectric perturbations are rapidly transduced into electrical signals, enabling highly sensitive, real-time, and label-free detection of specific biological targets [29]. During all measurements, the device operated under standardized settings: a sampling rate of 4 samples per second (4 SPS), eight parallel channels, and disposable SPEs consisting of a carbon working electrode and an Ag/AgCl reference electrode. The counter-electrode function was internally neutralized by the instrument’s circuitry and wireless data transmission was achieved via Bluetooth 4.0 for real-time monitoring [30].

Ballast water samples were provided by EMBIO Diagnostics’ industrial partner in the maritime sector, specialized in marine fuel supply and trading and operating in the port of Limassol (Cyprus). In all experiments, the SPEs operated in potentiometric (open-circuit potential) mode, detecting changes in electric potential (Volts) over time. In Phase 1, the electrodes recorded the intrinsic potentiometric signal of each ballast water sample applied directly onto the carbon working electrode. In Phase 2, the electrodes recorded the bioelectric response generated by the membrane-engineered Vero cells after exposure to the sample. All recordings consisted of time-series voltage data acquired at 4 SPS. More accurately, for the first experimental phase, 40 μL of each sample were applied directly to the eight channels of the electrodes, and measurements were recorded for one minute. For the second experimental phase, 20 μL of the biosensors was added on the electrode’s channels and then 20 μL of the sample suspension was added on top of the biosensor’s drop. Contaminated and non-contaminated ballast water samples were compared, and the resulting data were analyzed to identify microorganism concentrations that produced significant differences in electric potential. An algorithm was then developed to classify samples as either within or exceeding regulatory limits, providing an initial assessment of ballast water quality.

### 2.6. Data Processing and Algorithm Development

Each experiment produced a time series consisting of 720 voltage measurements per sample. Data processing was performed in Python (version 3.9) following the analytical framework described by Hadjilouka et al. [29]. The analysis involved two main stages, from which four feature vectors were derived. In total, 18 characteristic parameters were extracted and used to develop an algorithm for sample discrimination. In brief, these 18 parameters correspond to the features extracted from the cleaned potentiometric time-series data recorded by the 8 electrode channels. For each channel, two parameters were calculated: (i) the mean value of the cleaned signal and (ii) the minimum rolling-average value (window size 50). These two features were also computed once more across all 8 channels combined (overall mean and overall minimum rolling average). Thus, the final feature vector consisted of 2 parameters × 8 channels + 2 overall parameters (18 parameters) used for sample discrimination and algorithm development.

The results obtained from contaminated and non-contaminated samples were statistically compared using one-way analysis of variance (ANOVA) applied to the final processed output of each sample, following feature extraction and vector construction. This statistical comparison was used to identify significant differences (*p* < 0.05) between groups, from which the limit of detection (LOD) and threshold values distinguishing positive from negative samples were determined. Once these thresholds were established, the system automatically classified each new measurement as above or below the LOD in real time.

Finally, the performance of the newly developed method was evaluated in both phases by comparing its results with those obtained using traditional culture-based techniques. The parameters used to assess the method’s performance included sensitivity (Se: the ability of a test to correctly classify a sample as positive), specificity (Sp: the percentage of negative samples correctly identified by the test), positive predictive value (PPV: the probability that a sample is positive given a positive test result), and negative predictive value (NPV: the probability that a sample is negative given a negative test result).

## 3. Results

The objective of this study was to develop a rapid method for detecting *E. coli* and intestinal *Enterococci* and *V. cholerae* in BW prior to uptake, providing crews with an early warning system to identify microbial concentrations exceeding regulatory thresholds, in line with IMO standards. To achieve this, two experimental approaches were followed. The first involved direct potentiometric measurements of contaminated and non-contaminated samples, which demonstrated the feasibility of distinguishing microbial presence but were limited by background interference and relatively high detection thresholds. To overcome these limitations, a second approach was implemented using biosensors specifically engineered for these microorganisms. This strategy aimed to enhance specificity and lower the limit of detection, thereby allowing more reliable identification of microbial loads close to regulatory thresholds. Since detection of *V. cholerae* was not pursued beyond preliminary trials, no results for this bacterium will be presented in the current study.

**Figure 3:**
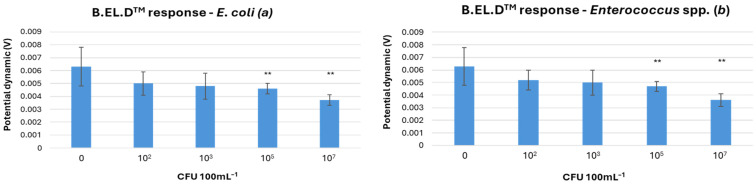
(**a**) Biosensor response to final *E. coli* concentrations of 10^2^, 10^3^, 10^5^, and 10^7^ CFU 100 mL^−1^, including a control. (**b**) Biosensor response to final *Enterococcus* spp. concentrations of 10^2^, 10^3^, 10^5^, and 10^7^ CFU 100 mL^−1^, including a control. Error bars represent the standard error of the mean from all replicates. Statistically significant differences in redox potential between the samples are indicated by asterisks (*p*-value < 0.05).

B.EL.D^TM^ Response to Varying Concentrations of *E. coli* and *Enterococcus* spp.: The measured electric potential in response to varying *E. coli* concentrations is shown in Figure 3a. The LOD in this approach was defined at 10^5^ CFU 100 mL^−1^ for *E. coli*, *Enterococcus* spp., and their combined presence. For *E. coli*, 71 experiments were conducted, of which 41 yielded successful detections. The dataset included 3 false positives, 18 true negatives, and 9 false negatives. Overall, the method achieved 84.5% accuracy, 84% sensitivity, 85.7% specificity, a positive predictive value (PPV) of 93.3%, and a negative predictive value (NPV) of 69.2%. For *Enterococcus* spp., a total of 78 experiments were conducted, of which 55 detected successfully the microorganism. There were 3 false positives, 18 true negatives, and 2 false negatives. Based on these results, the method demonstrated an accuracy of 93.6%, a sensitivity of 96.5%, a specificity of 85.7%, a positive predictive value of 94.8%, and a negative predictive value of 90% (Table 1). The potential dynamics obtained from the B.EL.D^TM^ device showed that at higher concentrations (10^5^ and 10^7^ CFU 100 mL^−1^), both *E. coli* and *Enterococcus* spp. were clearly distinguishable from negative controls (Figure 3). At lower concentrations, significant overlap with control samples was observed, likely due to naturally occurring background microorganisms in seawater. This interference, combined with the limited specificity of the basic potentiometric method, reduced detection clarity at low microbial levels.

**Figure 4:**
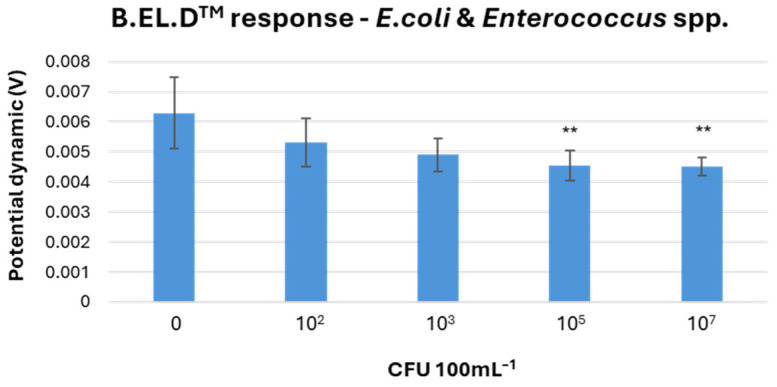
Biosensor response to final *E. coli* and *Enterococcus* spp. concentrations of 10^2^, 10^3^, 10^5^, and 10^7^ CFU 100 mL^−1^, including a control. Errors bars show the standard errors of the mean value of all replications. Statistically significant differences in redox potential between the samples are indicated by asterisks (*p*-value < 0.05).

A total of 75 experiments were then carried out using mixed samples containing both *E. coli* and *Enterococcus* spp., of which 53 yielded successful detection of the microorganisms. At higher concentrations, the microorganisms were again clearly differentiated from negative controls (Figure 4), whereas overlap at lower levels followed the same pattern observed previously. The results included 5 false positives, 16 true negatives, and 1 false negative. Overall, the method achieved 92% accuracy, 98.1% sensitivity, 76.1% specificity, a PPV of 91.3%, and an NPV of 94.1% (Table 1).

**Figure 5:**
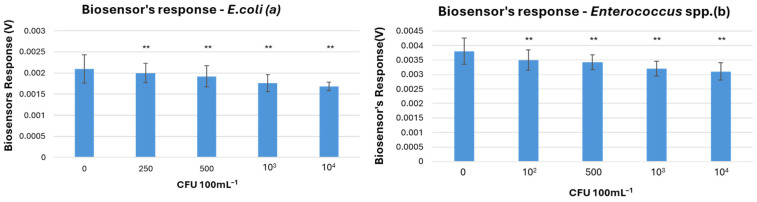
Biosensor response in presence of (**a**) *E. coli* and (**b**) *Enterococcus* spp. in ballast water samples. Statistically significant differences in redox potential between the samples are indicated by asterisks (*p*-value < 0.05).

Biosensor’s Response to Varying Concentrations of *E. coli* and *Enterococcus* spp.: Following the initial experiments with direct potential measurements, specialized biosensors were developed to enable specific detection of each microorganism at lower concentrations. The dynamic changes in potential measured in relation to microorganism concentration are presented in Figure 5. In this approach, the LOD was finally set at 250 CFU 100 mL^−1^ for *E. coli* and 10^2^ CFU 100 mL^−1^ for *Enterococcus* spp., in accordance with IMO regulatory standards.

For *E. coli*, a total of 67 experiments were performed. When the LOD was set at 250 CFU 100 mL^−1^, the method correctly detected 44 cases, with 5 false positives, 13 true negatives, and 5 false negatives. The corresponding performance metrics were 85.1% accuracy, 89.8% sensitivity, 72.2% specificity, 89.8% PPV, and 72.2% NPV. Similar results were obtained when the detection threshold was increased to 500 CFU 100 mL^−1^. At higher microbial concentrations (10^3^ and 10^4^ CFU 100 mL^−1^), the method clearly differentiated positive from negative samples, achieving excellent accuracy (92% and 97%, respectively) and robust performance characteristics (Table 2).

As shown in Figure 5b, and consistent with the results for *E. coli*, the biosensor’s performance for *Enterococcus* spp. was also satisfactory, showing reliable detection even at lower concentrations (10^2^ CFU 100 mL^−1^). A total of 72 experiments were conducted, and at an LOD of 10^2^ CFU 100 mL^−1^, the method achieved 81.9% accuracy, 83.3% sensitivity, 80% specificity, 85.4% PPV, and 77.4% NPV. Comparable results were observed at 500 CFU 100 mL^−1^, while the highest accuracy (92.2% and 97.4%) and discrimination ability were obtained at elevated concentrations (10^3^ and 10^4^ CFU 100 mL^−1^), as summarized in Table 2.

## 4. Discussion

Two complementary experimental approaches were applied in this study to assess the feasibility of a rapid, crew-operated method for evaluating the microbiological quality of BW. The first approach focused on direct potentiometric measurements of both contaminated and non-contaminated samples. The goal was to determine whether simple electrical potential readings could serve as a quick onboard alert system, allowing ship crews to identify potentially non-compliant water and initiate re-treatment before discharge. The findings showed that samples containing microorganisms could indeed be distinguished from clean ballast water. However, the method’s LOD (10^5^ CFU 100 mL^−1^) was high compared to the limits set by IMO. This outcome was expected, as the approach did not incorporate any microorganism-specific biorecognition component.

To address this limitation, a second, biosensor-based approach was developed, specifically targeting *E. coli* and *Enterococcus* spp. (i.e., *E. faecium* and *E. faecalis*). By integrating antibodies into the Vero cell’s membranes and utilizing these biosensors, the system achieved selective recognition of each microorganism, significantly improving both sensitivity and specificity. As a result, the LOD was reduced to IMO-relevant levels 250 CFU 100 mL^−1^ for *E. coli* and 10^2^ CFU mL^−1^ for *Enterococcus* spp. The substantial decrease in the detection limits observed in Phase 2 can be directly attributed to the use of membrane-engineered Vero cells carrying microorganism-specific antibodies. These biosensors introduce a selective biorecognition interface, enabling the device to distinguish target microorganisms from background microbiota, something that was not achievable with direct potentiometric measurements alone. This mechanistic contribution explains the marked improvement in analytical performance and further highlights the central role of the antibody-engineered cells in the enhanced sensitivity and specificity of the method. Together, these two methods represent a clear technological progression: from a general, rapid alert mechanism to a targeted, regulation-compliant detection system.

Recent advances in microfluidic and impedance-based detection technologies have further driven the field forward. For example, dielectrophoretic lab-on-chip platforms have detected approximately 69 ± 33 *E. coli* and 95 ± 34 *Enterococcus* cells within ten minutes in simulated BW [31]. Moreover, studies using potentiometric and phage-based sensors support the robustness of cell sensor interface mechanisms for detecting *E. coli* in aquatic matrices [32]. These results highlight the analytical power of such technologies; however, their reliance on controlled laboratory environments, delicate instrumentation, and complex fluid handling currently limits their deployment onboard ships. At the same time, the performance of existing commercial ballast water test kits underscores the importance of developing more advanced solutions. Although portable systems, they remain largely semi-quantitative, time-intensive, and influenced by operator expertise and environmental variability. This highlights the ongoing need for rapid, sensitive, and reliable onboard diagnostic technologies capable of delivering immediate and quantitative results.

The B.EL.D™ platform presented in this study offers a simpler and more robust alternative for shipboard applications. Its solid-state potentiometric design requires no flow control, optics, or sample labeling, while its Bluetooth-enabled interface allows real-time result visualization on a smartphone or tablet. The device functions reliably under typical shipboard conditions and vibration, salinity, and temperature fluctuations, and can be operated by minimally trained personnel. This makes it particularly suitable for rapid pre-discharge verification, helping crews ensure compliance with IMO D-2 standards [7], avoid costly port penalties and support proactive environmental protection.

Despite its clear advantages and strong analytical performance, some aspects of the system can be further optimized. The current biosensor design employs living cells as the biorecognition element, a configuration that has proven effective but naturally imposes a specific operational lifespan. This characteristic presents practical challenges for long-term storage and continuous usability, particularly in shipboard settings where diagnostic kits must remain ready for deployment during extended voyages far from port, with restricted access to replenishment. Moreover, detection of *V. cholerae* was not pursued beyond preliminary trials, as IMO regulations require its complete absence in 100 mL samples. This regulatory zero-tolerance standard makes single-cell detection impractical with field-deployable biosensors. This limitation reflects the stringent requirement rather than a technical failure of the method, underscoring the need for complementary approaches for such pathogens. Moreover, this study focused on ATCC reference strains because these are the internationally accepted model organisms used for validation of ballast water testing technologies. They provide consistent growth behavior and regulatory relevance. Nonetheless, future work will include additional *E. coli* and *Enterococcus* environmental isolates to further assess strain-to-strain variability.

Building on the success of the existing platform, future research will focus on enhancing its robustness and durability. A key direction involves functionalizing and immobilizing antibodies directly onto the sensor’s electrode surfaces, a modification expected to preserve the system’s high sensitivity while significantly improving its stability, shelf life, and overall performance under real maritime conditions. Optimization of antibody density will also be explored to maximize signal-to-noise ratios and fine-tune detection efficiency, including revisiting *Vibrio cholerae* detection using lower or higher antibody concentrations. In parallel, additional improvements are being pursued, such as integrating microfluidic pre-concentration modules to increase the effective bacterial load delivered to the biorecognition interface and incorporating short enrichment steps to further enhance sensitivity. Ongoing work aims to combine these advances into a next-generation platform with regulatory-level performance. Under these improved conditions, evaluating LOD values below the IMO thresholds will become scientifically meaningful and will be incorporated in subsequent validation phases.

## 5. Conclusions

This study developed and validated a rapid, portable method for detecting *Escherichia coli* and *Enterococcus* spp. in ballast water, enabling real-time onboard assessment of microbial compliance with IMO D-2 standards. Two complementary approaches were applied: direct potentiometric measurements for rapid screening and microorganism-specific biosensors integrated into the B.EL.D™ platform for enhanced sensitivity and specificity. The biosensor-based system achieved detection limits of 250 CFU 100 mL^−1^ for *E. coli* and 10^2^ CFU 100 mL^−1^ for *Enterococcus* spp., demonstrating high accuracy and reliability under simulated field conditions. Compared with conventional and molecular techniques, the B.EL.D™ platform offers faster analysis, minimal sample preparation, and user-friendly operation without laboratory infrastructure. Future improvements will focus on enhancing performance at lower microbial concentrations and expanding detection to additional indicator species. Overall, the B.EL.D™ biosensor provides a practical, regulation-compliant tool for autonomous ballast water monitoring and sustainable maritime operations.

## Figures and Tables

**Table 1:** Performance characteristics of the B.EL.D^TM^ device in presence of *E. coli*, *Enterococcus* spp., and their combination in ballast water samples, with LOD 10^5^ CFU 100 mL^−1^.

Indices	*E. coli*	*Enterococcus* spp.	*E. coli* and *Enterococcus* spp.
Accuracy	84.5%	93.6%	92.0%
Sensitivity	84.0%	96.5%	98.1%
Specificity	85.7%	85.7%	76.1%
Positive Predictive Value	93.3%	94.8%	91.3%
Negative Predictive Value	69.2%	90.0%	94.1%

**Table 2:** Performance characteristics of the B.EL.D™ biosensor for the detection of *E. coli* and *Enterococcus* spp. at different microbial concentrations (expressed in CFU 100 mL^−1^). The table summarizes accuracy, sensitivity, specificity, PPV, and NPV for each tested detection threshold.

Indices	*E. coli* (CFU 100 mL^−1^)				*Enterococcus* spp. (CFU 100 mL^−1^)			
	250	500	10^3^	10^4^	10^2^	500	10^3^	10^4^
Accuracy	85.1%	84%	92%	97%	81.9%	83.6%	92.2%	97.4%
Sensitivity	89.8%	90%	97%	100%	83.3%	83.9%	85.7%	100%
Specificity	72.2%	72%	83%	94%	80%	83.3%	96.7%	96.7%
PPV	89.8%	88%	91%	95%	85.4%	83.9%	94.7%	90%
NPV	72.2%	76%	94%	100%	77.4%	83.3%	90.6%	100%

## Data Availability

The data presented in this study are not publicly available due to legal and intellectual property restrictions, as they are proprietary to the company involved. Data may be made available from the corresponding author upon reasonable request and with permission of the data owner.

## References

[1] Bradie J., Rolla M., Bailey S.A., MacIsaac H.J. (2023). Managing Risk of Non-Indigenous Species Establishment Associated with Ballast Water Discharges from Ships with Bypassed or Inoperable Ballast Water Management Systems. J. Appl. Ecol..

[2] (2011). Ballast Water. Guide to Ship Sanitation.

[3] Kurniawan S.B., Pambudi D.S.A., Ahmad M.M., Alfanda B.D., Imron M.F., Abdullah S.R.S. (2022). Ecological Impacts of Ballast Water Loading and Discharge: Insight into the Toxicity and Accumulation of Disinfection by-Products. Heliyon.

[4] Lv B., Zhu G., Tian W., Guo C., Lu X., Han Y., An T., Cui Y., Jiang T. (2023). The Prevalence of Potential Pathogens in Ballast Water and Sediments of Oceangoing Vessels and Implications for Management. Environ. Res..

[5] Zatoń-Sieczka K., Czerniejewski P. (2024). The Impact of Microorganisms Transported in Ships’ Ballast Water on the Fish of the Estuarine Waters and Environmental Sustainability in the Southern Baltic Sea. Sustainability.

[6] Cha H.-G., Hyun B., Jang M.-C., Choi K.-H., Shin K., Seo J.-Y., Jang P.-G. (2024). Simulated Testing of the Characteristics and Environmental Impacts of Disinfection By-Products Generated by Ballast Water Management Systems in Ports during Phytoplankton Blooms. J. Mar. Sci. Eng..

[7] (2004). International Convention for the Control and Management of Ships’ Ballast Water and Sediments (BWM) (Regulation D-2). Ballast Water Performance Standard.

[8] Čampara L., Frančić V., Maglić L., Hasanspahić N. (2019). Overview and Comparison of the IMO and the US Maritime Administration Ballast Water Management Regulations. J. Mar. Sci. Eng..

[9] Outinen O., Bailey S.A., Casas-Monroy O., Delacroix S., Gorgula S., Griniene E., Kakkonen J.E., Srebaliene G. (2024). Biological Testing of Ships’ Ballast Water Indicates Challenges for the Implementation of the Ballast Water Management Convention. Front. Mar. Sci..

[10] Hess-Erga O.-K., Moreno-Andrés J., Enger Ø., Vadstein O. (2019). Microorganisms in Ballast Water: Disinfection, Community Dynamics, and Implications for Management. Sci. Total Environ..

[11] Deblais L., Ahmedo B.U., Ojeda A., Mummed B., Wang Y., Mekonnen Y.T., Demisie Weldesenbet Y., Hassen K.A., Brhane M., McKune S. (2025). Assessing Fecal Contamination from Human and Environmental Sources Using *Escherichia coli* as an Indicator in Rural Eastern Ethiopian Households—A Cross-Sectional Study from the EXCAM Project. Front. Public Health.

[12] Alonzo L.F., Jain P., Hinkley T., Clute-Reinig N., Garing S., Spencer E., Dinh V.T.T., Bell D., Nugen S., Nichols K.P. (2022). Rapid, Sensitive, and Low-Cost Detection of Escherichia Coli Bacteria in Contaminated Water Samples Using a Phage-Based Assay. Sci. Rep..

[13] Said M.S., Tirthani E., Lesho E. (2025). Enterococcus Infections. StatPearls.

[14] Ramamurthy T., Mutreja A., Weill F.-X., Das B., Ghosh A., Nair G.B. (2019). Revisiting the Global Epidemiology of Cholera in Conjunction with the Genomics of Vibrio Cholerae. Front. Public Health.

[15] Fournier J.-M., Quilici M.-L. (2007). Choléra. Presse Médicale.

[16] National Research Council (US) Committee on Indicators for Waterborne Pathogens (2004). New Biological Measurement Opportunities. Indicators for Waterborne Pathogens.

[17] Kabiraz M.P., Majumdar P.R., Mahmud M.M.C., Bhowmik S., Ali A. (2023). Conventional and Advanced Detection Techniques of Foodborne Pathogens: A Comprehensive Review. Heliyon.

[18] Joachimsthal E.L., Ivanov V., Tay J.-H., Tay S.T.-L. (2003). Flow Cytometry and Conventional Enumeration of Microorganisms in Ships’ Ballast Water and Marine Samples. Mar. Pollut. Bull..

[19] Pernice M.C., Gasol J.M. (2023). Automated Flow Cytometry as a Tool to Obtain a Fine-Grain Picture of Marine Prokaryote Community Structure along an Entire Oceanographic Cruise. Front. Microbiol..

[20] Fykse E.M., Nilsen T., Nielsen A.D., Tryland I., Delacroix S., Blatny J.M. (2012). Real-Time PCR and NASBA for Rapid and Sensitive Detection of *Vibrio cholerae* in Ballast Water. Mar. Pollut. Bull..

[21] Fernández Blanco A., Moreno Y., García-Hernández J., Hernández M. (2024). A Photonic Immunosensor Detection Method for Viable and Non-Viable E. Coli in Water Samples. Microorganisms.

[22] Maw M.M., Pan X., Peng Z., Wang Y., Zhao L., Dai B., Wang J. (2018). A Changeable Lab-on-a-Chip Detector for Marine Nonindigenous Microorganisms in Ship’s Ballast Water. Micromachines.

[23] MICROBIOLOGICAL DIPSLIDES DTK Water Test Kits—Simplified Test Water Analysis. https://www.droptestkits.com/microbiology/.

[24] Insatech, AVS Parker Kittiwake Density Meter-Accuracy-like Laboratory Tests. https://www.insatechmarine.com/products/water-test-treatment/water-test-kits/ballast-water-test-kit/#product-info.

[25] Scan VIT® Ballast Water Enterococcus/*E. coli*. https://www.vermicon.com/products/scanvit-water/test-kit-quantitative-analysis-balast-water-enterococcus-ecoli.

[26] Luminultra BQUA Plus Test Kit for Ballast Water. https://www.luminultra.com/product/bqua-plus-test-kit-for-ballast-water/.

[27] Apostolou T., Loizou K., Hadjilouka A., Inglezakis A., Kintzios S. (2020). Newly Developed System for Acetamiprid Residue Screening in the Lettuce Samples Based on a Bioelectric Cell Biosensor. Biosensors.

[28] Kintzios S., Pistola E., Panagiotopoulos P., Bomsel M., Alexandropoulos N., Bem F., Ekonomou G., Biselis J., Levin R. (2001). Bioelectric Recognition Assay (BERA). Biosens. Bioelectron..

[29] Hadjilouka A., Loizou K., Apostolou T., Dougiakis L., Inglezakis A., Tsaltas D. (2020). Newly Developed System for the Robust Detection of Listeria Monocytogenes Based on a Bioelectric Cell Biosensor. Biosensors.

[30] Konstantinou L., Varda E., Hadjilouka A., Loizou K., Dougiakis L., Inglezakis A., Attipa C., Papazoglou I., Apostolou T. (2024). Redox Potential Analysis for Activated Carbon Using B.EL.D^TM^ Technology: A Novel Application. Sens. Bio-Sens. Res..

[31] Kong T.F., Shen X., Marcos, Yang C., Ibrahim I.H. (2020). Dielectrophoretic Trapping and Impedance Detection of *Escherichia coli*, Vibrio Cholera, and Enterococci Bacteria. Biomicrofluidics.

[32] Maw M.M., Wang J., Li F., Jiang J., Song Y., Pan X. (2015). Novel Electrokinetic Microfluidic Detector for Evaluating Effectiveness of Microalgae Disinfection in Ship Ballast Water. Int. J. Mol. Sci..

